# MicroRNA-138 promotes neuroblastoma SH-SY5Y cell apoptosis by directly targeting DEK in Alzheimer’s disease cell model

**DOI:** 10.1186/s12868-020-00579-z

**Published:** 2020-07-31

**Authors:** Jin Miao, Jin Jing, Yixiang Shao, Huaichang Sun

**Affiliations:** 1 College of Veterinary Medicine, Yangzhou University, Yangzhou, 225009 Jiangsu People’s Republic of China; 2 Laboratory Animal Center, Nantong University, Nantong, 226000 Jiangsu People’s Republic of China

**Keywords:** miR-138, DEK, AKT, Apoptosis, Alzheimer’s disease

## Abstract

**Background:**

Alzheimer’s disease (AD) is a progressive neuro-degenerative disease with a major manifestation of dementia. MicroRNAs were reported to regulate the transcript expression in patients with Alzheimer’s disease (AD). In this study, we investigated the roles of miR-138, a brain-enriched miRNA, in the AD cell model.

**Methods:**

The targets of miRNA-138 was predicted by bioinformatic analysis. The expression levels of DEK at both mRNA and protein levels were determined by qRT-PCR and Western blot, respectively. Luciferase assays were carried out to examine cell viabilities. Hoechst 33258 staining was used to detect cell apoptosis.

**Results:**

Our results demonstrated that the expression levels of miR-138 were increased in AD model, and DEK was a target of miR-138. Overexpression of miR-138 in SH-SY5Y cells obviously down-regulated the expression of DEK in SH-SY5Y cells, resulting in the inactivation of AKT and increased expression levels of proapoptotic caspase-3. MiR-138 mediated-suppression of DEK increased the susceptibility of cell apoptosis.

**Conclusions:**

MicroRNA-138 promotes cell apoptosis of SH-SY5Y by targeting DEK in SH-SY5Y AD cell model. The regulation of miR-138 may contribute to AD via down-regulation of the DEK/AKT pathway.

## Background

Alzheimer’s disease (AD) is a progressive neuro-degenerative disease with a major manifestation of dementia [11]. There are a large number of Alzheimer’s disease-related dementia patients all over the world, and they are usually accompanied by memory loss and behavioral changes, especially in older adults [26]. It has a complex progression involving neuronal dysplasia, angiogenic changes, and release of inflammatory mediators [33]. The early diagnosis of AD is complicated and laborious [13, 24]. Therefore, novel biomarkers and therapeutic agents have been investigated for use in patient stratification. Non-coding RNAs have been demonstrated to be potential biomarkers and diagnostic tools for many diseases [21, 30].

Excessive apoptosis of cells might be the cause of neurodegenerative disease in AD patients. MicroRNAs (miRNAs) are small non-coding RNAs with molecular regulation functions in protein metabolism, cell apoptosis, and many other neurobiological processes [28]. Their functions in cell apoptosis show great potentials as regulating factors to alleviate cell death for AD patients. Previous studies have shown that the dysregulation of miRNA is in correlation with some types of neurodegenerative disease, and they are also involved in the regulation of some Alzheimer’s disease-related proteins [16, 27]. Various miRNAs have been reported to be related with Alzheimer’s disease, such as miR-29a/b-1 [9], miR-107 [32], miR-34a [34], miR-122 [14], miR-455-3p [15, 17] and miR-26b [1]. It was reported that miR-26b was obviously up-regulated in Alzheimer’s Disease, in which miR-26b participated in the activation of cycle entry, tau-phosphorylation, and postmitotic neuron apoptosis [1]. MiR-138, a key member of miRNAs, was reported to promote tau phosphorylation via the targeting of retinoic acid receptor alpha [35]. However, the regulation of miR-138 in AD progression is still unknown. In the present study, we aimed to investigate the regulation of miR-138 in the clinical pathology of Alzheimer’s disease.

DEK is a highly conserved ubiquitous oncogene in the proliferating cells of all tissues, which is regulated at both transcriptional and post-translational levels in DNA repair, replication, transcriptional regulation and mRNA splicing [19, 31]. Emerging evidence has suggested that the overexpression of DEK could inhibit cell death, and the silencing of DEK leads to cell apoptosis via stabilization and transcriptional activation of p53 [2, 38]. AKT, a serine/threonine kinase that has a wide range of substrates, is involved in the regulation of many biological processes, such as cell proliferation, cell growth and apoptosis [12, 23]. The role of DEK and AKT in cellular survival suggests their functional mechanisms in Alzheimer’s disease [20].

β-amyloid (Aβ), a cleavage product of the amyloid precursor protein (APP), is the main component of insoluble senile plaques and its deposition is neurotoxic and can induce apoptosis of neurons [21]. In this study, we focused on exploring the inner associations of miR-138, DEK oncogene and AKT, and other factors that lead to apoptosis in AD cell model, which was established using Aβ1-42 in SH-SY5Y cells.

## Materials and methods

### Antibodies and reagents

Rabbit-anti-DEK antibody was obtained from Abcam, UK. Anti-Rabbit Cleaved Caspase-3 (Asp175) antibody, anti-Caspase-3 (8G10) antibody, anti-pAKT (ser473) antibody, anti-AKT antibody were obtained from CST, USA. HA-Tag (26D11) mouse antibody was obtained from AbMart. Mouse anti-GAPDH antibody was obtained from Sigma-Aldrich, USA. HRP-conjugated goat anti-mouse IgG and goat anti-rabbit IgG were obtained from Santa Cruz Biotechnology, USA. The ApopTag^®^ kit were purchased from Millipore, USA. Aβ1-42 peptide (Sigma, USA) was dissolved in sterile distilled water at a concentration of 1 mM and stored at − 20 °C. Cells were transfected with equal amounts of miRNA-138 mimic and/or miRNA-138 inhibitor for 48 h, and then treated with 20 μM Aβ42 for 24 h.

### Cell culture and transfection

SH-SY5Y cell lines were cultured in DMEM with 10% FBS and 100 U/ml penicillin/streptomycin (ATCC, USA) in a humidified environment at 37 °C with 5% CO_2_. The cells were kept and seeded with a ratio of 1:5. The culture media was replaced every 4 days. The mature miR-138 sequences were obtained from the miRNA registry. The sequences of ASO miR-138 were designed, according to the principle of sequences complementary to the mature miR-138. Mature miR-138, ASO miR-138 and short-hairpin RNA directed against DEK (sense 5′-GGUGUGCACUGUGAGAUCAtt-3′, antisense 5′-UGAUCUCACAGUGCACACCct-3′) were constructed using pcDNA3.1 plasmid as the backbone (GenePharma, China). Lipofectamine-3000 (Invitrogen) was used to transfect the cells (Invitrogen, USA).

### Luciferase assay

The WT or MUT 3′-UTR luciferase reporter plasmids, as well as a miR-138 mimic or miR-138 inhibitor, were co-transfected to SH-SY5Y cells. Cells were harvested at 48 h after transfection and the Dual-Luciferase Reporter Assay (Promega, WI, USA) was performed.

### RNA isolation and quantitative RT-PCR

Total RNAs were extracted using TRIZOL (Invitrogen, USA). For miRNA analysis, the TaqMan microRNA Reverse Transcription reagents and the Universal PCR Master Mix with microRNA real-time PCR primers (Applied Biosystems) were used. Total RNAs (1 μg) were reverse transcribed to cDNAs. For mRNA expression analysis, 1 μg of total RNAs were reverse-transcribed with the TaqMan Reverse Transcription Reagents (Applied Biosystems N808-0234). Gene expression was measured by SYBR green (Applied Biosystems, USA). U6 and GAPDH were used as endogenous control. The primers used were listed in Table 1.

**Table 1:** Sequences of primers used in qRT-PCR

Name	Forward primer (5′-3′)	Reversed primer (5′-3′)
miR-138	AGCUGGUGUUGUGAAUC	GTGCAGGGTCCGAGGT
U6	GTAGTCGGCGAAGGTCTCAC	ACCGTGGATGCAATGCCTAA
DEK	AAAGCCACCTACAGATGAAGAG	TCCTCTCAGTCAAATCACAAGC
GAPDH	GGTTGTCTCCTGCGACTTCA	GGTGGTCCAGGGTTTCTTACTC

### Western blot

Cells were washed and lysed in buffer (50 mM Tris–HCl pH 8.0, 150 mM NaCl, 1% NP-40, 0.5% sodium deoxycholate, 0.1% SDS) with protease inhibitor cocktail (Roche, Switzerland) and 1 mM phenylmethylsulfonyl-fluoride for 30 min. Extracts were centrifuged at 14,000×*g* for 30 min and concentrations were measured using a BCA Protein Quantitative Analysis Kit (Biocolors, Shanghai, China). A total of 20 mg lysates was boiled, separated by SDS-PAGE and transferred to PVDF membranes. The membranes were blocked using 5% skim milk for 1 h and then incubated at 4 °C for overnight with anti-DEK (1:1000), anti-AKT (1:1000), anti-p-AKT (1:1000), anti-Cleaved Caspase-3 (1:1000), and anti-Caspase-3 (1:1000). After washing, membranes were incubated with HRP-conjugated goat anti-mouse antibody (1:5000) at 25 °C for 2 h. Western blot was carried out by Immobilon Western Chemiluminescent HRP Substrate (Millipore, USA) and quantified by Image J. All experiments were conducted for three times.

### Flow cytometry

Cells were centrifuged and stained with Annexin V-FITC and propidium iodide (PI) using the Annexin V-FITC Apoptosis Detection Kit (BD Biosciences). Briefly, cells were re-suspended and 5 μl of Annexin V-FITC and 1 μl PI were added. Flow cytometry was conducted on a flow cytometer (Becton–Dickinson; LSR II) and apoptotic cell percentage was determined.

### Hoechst 33258 staining

Cell apoptosis was evaluated by Hoechst 33258 staining. After treatment, cells were harvested, washed, and fixed with 4% (v/v) paraformaldehyde at 25 °C for 30 min. Cells were washed, stained with 2 μl 5 mg/ml Hoechst 33258 and incubated for 10 min. Stained cells were washed and observed under a fluorescence microscope (Leica DMI-4000B, Germany). Cell apoptosis was calculated by DNA fragmentation and nuclear shrinkage. The apoptotic rate was calculated as several apoptotic cells/number of total cells (> 300) × 100.

### Co-immunoprecipitation (Co-IP)

Plasmids were transfected into SH-SY5Y cells using Lipofectamine 3000. Cell lysate was harvested at 48 h after transfection in non-denaturing lysis buffer containing 20 mM Tris HCl (pH 8.0), 137 mM NaCl, 10% glycerol, 1% Nonidet P-40, 2 mM EDTA and protease inhibitors. The supernatant was incubated with the primary antibody (1:500) and A+G Sepharose at 4 °C for 4 h. The beads were washed and reactivated by boiling in sample buffer. The samples were detected by Western blot.

### Statistical analysis

SPSS 19.0 was used for data analysis. Data were expressed as mean ± stand deviation (SD). T-test was used for comparisons between two groups. One-way ANOVA and Bonferroni’s post hoc test was used for exploring the differences among multi-groups. *P* < 0.05 was considered as statistically significant.

## Results

### The expression levels of miR-138 were increased in AD model

**Fig. 1:**
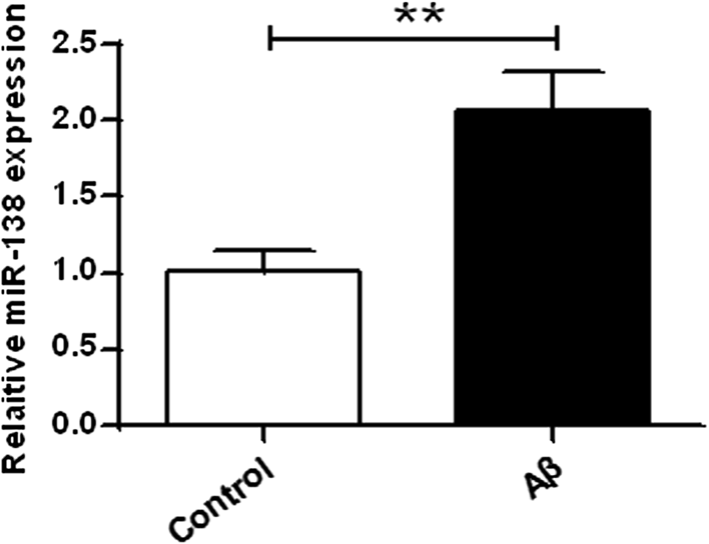
Expression of miR-138 was obviously higher in SH-SY5Y cells exposed to Aβ

qRT-PCR was carried to measure the expression levels of miR-138 in AD model. The expression of miR-138 was obviously upregulated in Aβ treated SH-SY5Y cells than that in the untreated (Control) group (*P* < 0.01) (Fig. 1). Therefore, it was confirmed that the expression of miR-138 was significantly upregulated in AD model.

### DEK was a target of miR-138

**Fig. 2:**
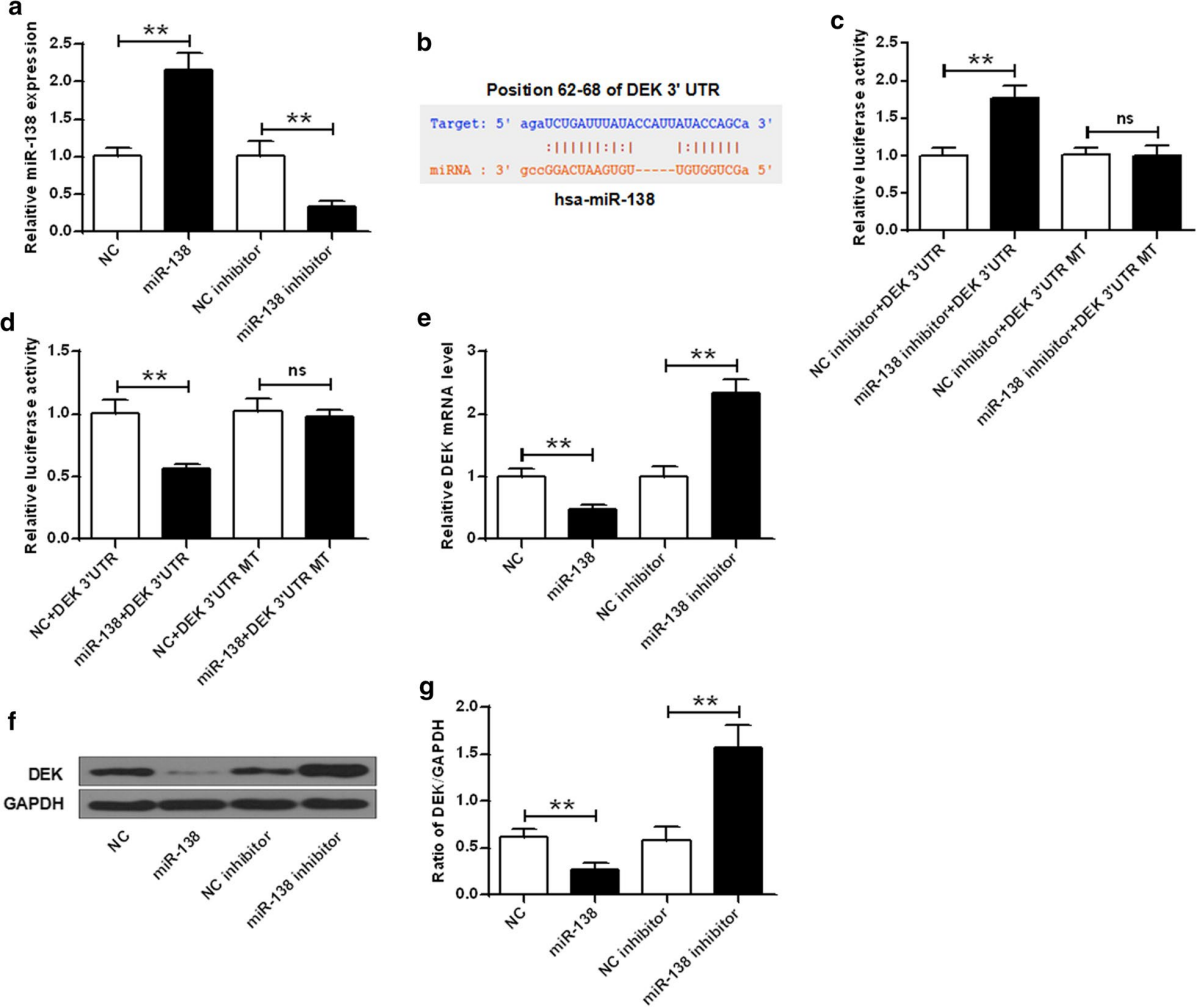
DEK was a direct target gene of miR-138. **a** qRT-PCR for miR-138 mRNA expression in SH-SY5Y cell model. **b** Bioinformatics analysis between DEK and miR-138. **c** Luciferase activity for cells transfected with miR-138 inhibitor or negative control and DEK 3′-UTR or DEK 3′-UTR MT. **d** Luciferase activity for cells transfected with miR-138 or negative control and DEK 3′-UTR or DEK 3′-UTR MT. **e** DEK mRNA expression after transfection with miR-138 or miR-138 inhibitor were detected by qRT-PCR. **f**, **g** DEK expression after transfection with miR-138 or miR-138 inhibitor were detected by Western blot (**P < 0.01)

Firstly, we identified the successful transfection of miR-138 by RT-PCR. The relative expression of miR-138 was remarkably elevated after transfection with miR-138 mimic (*P* < 0.01) and decreased after transfection with miR-138 inhibitor (*P* < 0.01) (Fig. 2a). The relationship between DEK and miR-138 was then evaluated by bioinformatics analysis and luciferase assay. DEK was predicted to be a target of miR-138 through bioinformatics analysis using TargetScan. org and microRNA.org (Fig. 2b). Luciferase reporter assay results demonstrated that the activity was greatly reduced after transfection with miR-138 and DEK 3′-UTR, but obviously elevated by miR-138 inhibitor and DEK 3′-UTR (*P* < 0.01) (Fig. 2c, d). In addition, the expression levels of DEK were significantly decreased by miRNA-138 mimic at both mRNA (Fig. 2e) and protein (Fig. 2f, g) levels, but was notably enhanced by miR-138 inhibitor (*P* < 0.01) (Fig. 2e–g). These data indicated that DEK was targeted by miR-138.

### DEK activated AKT phosphorylation (Ser473) while inhibited cleaved caspase-3 expression in SH-SY5Y cells

**Fig. 3:**
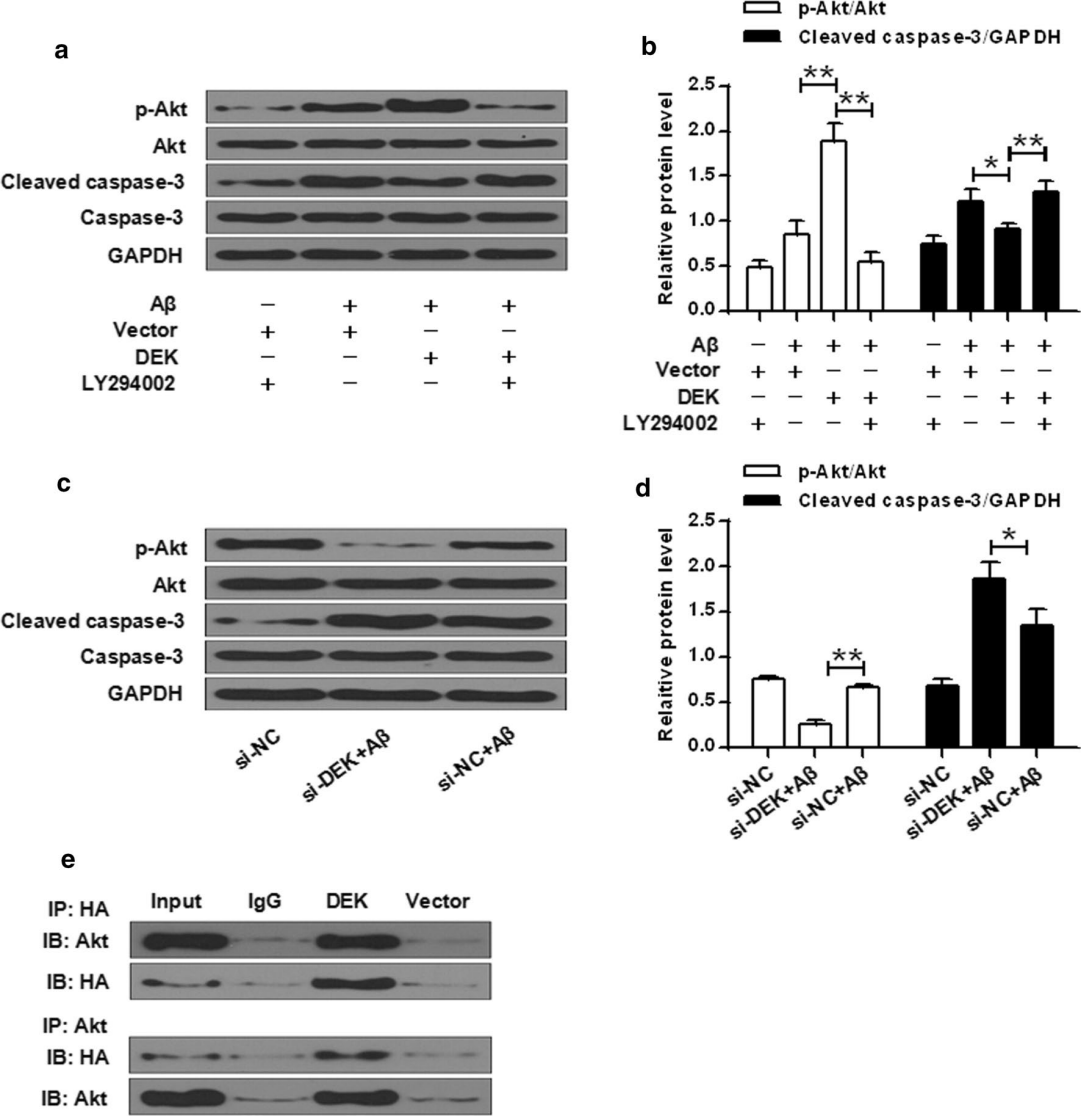
DEK activated AKT phosphorylation while inhibited cleaved caspase-3 expression in Aβ1-42 treated SH-SY5Y cells. Transfection with DEK leads to an increase in the level of p-Akt and a decrease in cleaved caspase-3 in Aβ42 treated SH-SY5Y cells. **a**, **b** p-Akt, total Akt, cleaved caspase-3, total caspase-3 and GAPDH levels were measured by western blot analysis and normalized to GAPDH, total Akt and total caspase-3, respectively. **c**, **d** Up regulation of cleaved caspase-3 and inhibition of p-Akt induced by transfection with si-DEK for DEK knockdown, indicates that DEK activates the Akt signaling pathway. **e** Co-IP was conducted using lysates from SHSY5Y cells transfected with AKT and DEK mini-receptor. Cell lysates were incubated with antibodies specific for Akt or DEK, or normal IgG, and the immunoprecipitated complexes were analyzed by western blot for Akt and DEK, as indicated. *P < 0.05, **P < 0.01

The role of DEK in AKT activation was investigated by western blot. DEK inhibited caspase-3 activity via activating the AKT signaling pathway in Aβ treated cells (*P* < 0.05, *P* < 0.01) (Fig. 3a, b). Silencing of DEK suppressed AKT phosphorylation and induced a change in cleaved caspase-3 activity (*P* < 0.05, *P* < 0.01) (Fig. 3c, d). Immunoprecipitated complexes were analyzed by Western blot for AKT and hemagglutinin (HA)-tagged DEK (Fig. 3e). The results suggested that the two molecules could interact with each other. These results demonstrated that DEK inhibited the expression of cleaved caspase-3 through the activation of the AKT signaling.

### MiR-138 mediated suppression of DEK increased susceptibility of cell apoptosis

**Fig. 4:**
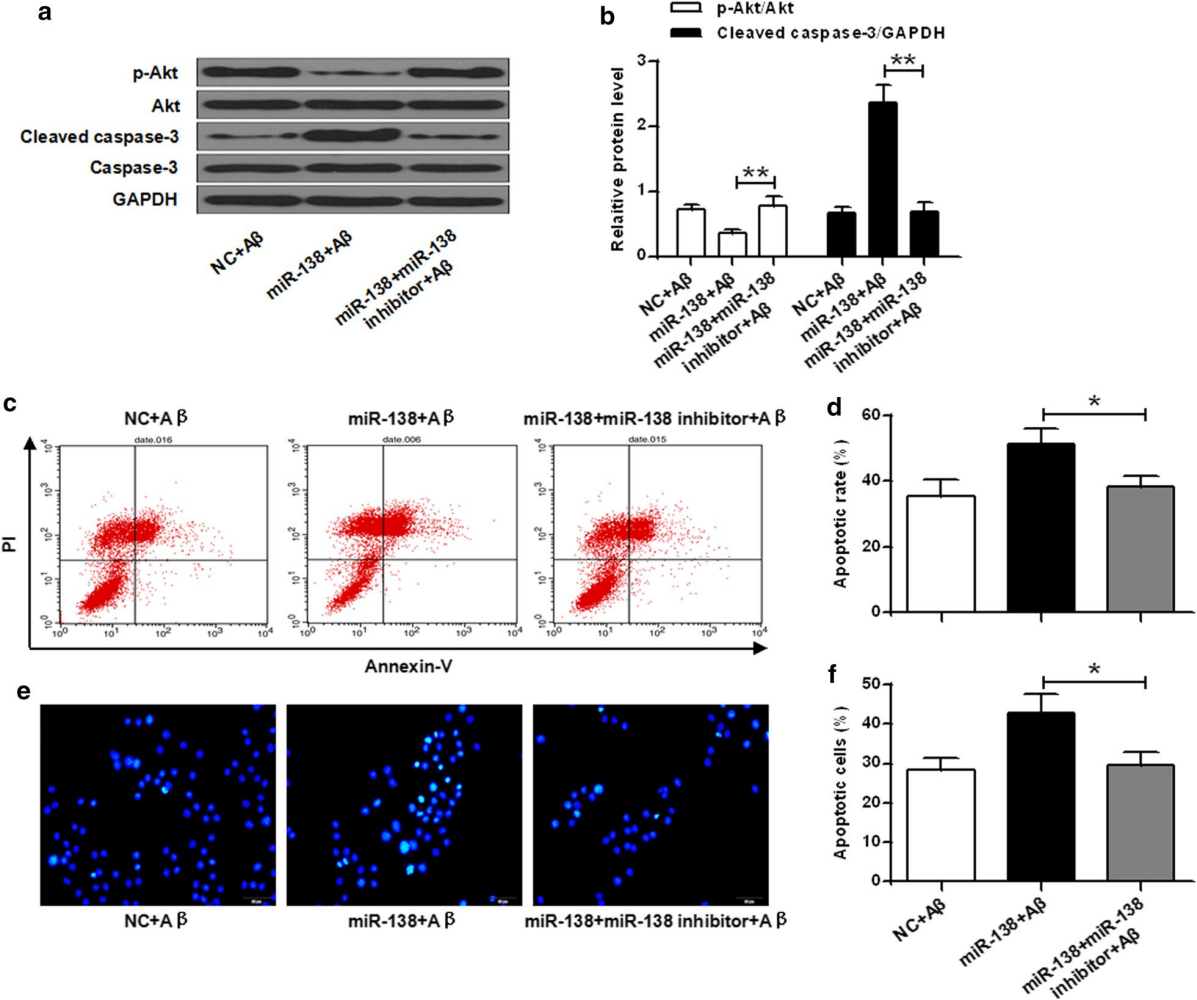
MiR-138 mediated suppression of DEK increased susceptibility of cell apoptosis. **a** Pre-treatment with miR-138 significantly decreased the level of p-Akt and upregulated the levels of cleaved caspase-3. **b** Quantitative analysis of p-Akt and cleaved caspase-3 protein levels from a, normalized to GAPDH protein level. **c** Effects of miRNA-138 on cell apoptosis induced by Aβ42 were assessed by flow cytometry. **d** Quantification of the percentage of apoptotic cells after exposure to Aβ42 in the presence of miRNA-138 mimic and/or miRNA-138 inhibitor. **e** Cell apoptosis observed by Hoechst 33258 staining using a fluorescence microscope (200×). Scale bar, 100 μm. **f** Percentage of apoptotic cells. *P < 0.05, **P < 0.01

Western blot was utilized to examine the effect of miR-138 in caspase-3 activity. Our results showed that miRNA-138 changed caspase-3 activity that was affected by Aβ treatment (*P* < 0.01) (Fig. 4a, b). The flow cytometry indicated that the cellular apoptosis was 35.42%, 51.40% and 38.26% with Aβ alone, Aβ plus miRNA-138 mimic and Aβ plus miRNA-138 inhibitor, respectively (*P* < 0.05) (Fig. 4c, d). Additionally, apoptotic cells in the miRNA-138 group were significantly higher than that in the negative control (*P* < 0.05) (Fig. 4e, f). These results indicated that miRNA-138 induced increased cell apoptosis in SH-SY5Y cells exposed to Aβ.

## Discussion

Dementia, a commonly known symptom of Alzheimer’s disease, results in disorientation, loss of memory, and visual-spatial abilities in elderly people [3]. Neuro-pathology in the brains exists in AD patients for years before these symptoms [10]. Therefore, it is critical to develop novel and practical biomarkers for early diagnosis of AD. Non-coding RNAs arise as new therapeutic agents for various human diseases, including neuro-defective diseases [30], cardiovascular diseases [18], and human cancers [7]. In this study, we investigated the role of miRNA-138 and its associations with oncogene DEK and AKT in the regulation of neuron apoptosis.

MiRNAs are small noncoding RNAs that can regulate gene expression at the post-transcriptional level via binding to 3′-UTR of the target gene [4]. Previous reports have identified several miRNAs that explicitly expressed in the brain cells, and some are involved in neuron differentiation, synaptic plasticity, and memory formation [20]. Several miRNAs have been reported to be related with Alzheimer’s disease, such as miR-29a/b-1 [9], miR-107 [32], miR-34a [34], miR-122 [14], miR-455-3p [15, 17], and miR-26b [1]. For example, it was noted that microRNA could regulate Alzheimer’s amyloid precursor protein expression [8]. Dysregulation of specific miRNAs was observed in Alzheimer’s disease [32]. The qRT-PCR results in this study revealed that the expression of miR-138 were remarkably upregulated in Aβ treated SH-SY5Y cells compared to that in the untreated cells. Our findings that the expression of miR-138 was elevated in AD cell model are in consistence with previous researches, indicating that miR-138 actually plays a regulatory role in AD progression.

The human DEK oncogene was first discovered as a fusion with the gene encoding the CAN nucleoporin protein in myeloid leukemia patients [6]. Up-regulated expression of DEK was related to many human diseases, such as uterine cervical cancer [36], gastric cancer [25], and neuroblastoma tumor [39]. Over-expression of DEK could inhibit cell death, and knockdown of DEK could result in cell apoptosis. It was reported that DEK was a target of miR-200a [37]. The bioinformatics analysis illustrates that DEK was a target of miR-138. The luciferase reporter assay demonstrates that the transcriptional activity in cells was substantially decreased after transfection with miR-138 and DEK 3′-UTR, but remarkably elevated by miR-138 inhibitor and DEK 3′-UTR. In addition, the mRNA and protein levels of DEK were greatly decreased by miRNA-138 mimic but was notably enhanced by miR-138 inhibitor. Our results further confirmed that DEK was a direct target gene of miR-138.

It was demonstrated that DEK markedly attenuated AKT phosphorylation [5]. Our qRT-PCR and western blot results revealed that DEK could inhibit caspase-3 activity via the AKT signaling pathway. In addition, silencing of DEK suppressed AKT phosphorylation and affected caspase-3 activity. The results of Co-IP for SH-SY5Y cells transfected with AKT and DEK mini-receptor also suggested that DEK inhibited the expression of cleaved caspase-3 through activation of AKT.

AKT signaling pathways play essential roles in the regulation of various cell functions such as nutrient metabolism, cell growth, apoptosis and survival [29]. The activation of the AKT pathway was reported to be involved with miRNA interactions [22]. In our experiments, miR-138 significantly decreased p-AKT and up-regulated the expression of cleaved caspase-3 protein.

## Conclusion

The results of flow cytometry and apoptotic test demonstrated that apoptotic cells in the miR-138 mimic group were much higher than that in the control. It indicated that miRNA-138 induced increased cell apoptosis in this AD cell model. Our observations further confirmed that the regulation of microRNA-138 may contribute to AD through down-regulation of DEK/AKT pathway.

## Data Availability

The analyzed data sets generated during the study are available from the corresponding author on reasonable request.

## Abbreviations

AD: Alzheimer’s disease
miRNAs: MicroRNAs
